# Off-pump resection of a giant inflamed epicardial cyst

**DOI:** 10.1186/s40792-018-0495-3

**Published:** 2018-08-06

**Authors:** Daisuke Kaneyuki, Tomoki Sakata, Anan Nomura, Manabu Sakurai, Kenji Mogi, Yoshiharu Takahara

**Affiliations:** Division of Cardiovascular Surgery, Heart and Vascular Institute, Funabashi Municipal Medical Center, 1-21-1 Kanasugi, Funabashi-shi, Chiba, 273-8588 Japan

**Keywords:** Epicardial cyst, Pericardial cyst, Off-pump surgery, Video-assisted thoracoscopic surgery

## Abstract

**Background:**

Epicardial cysts are rarer benign tumors than pericardial cysts. There have been few reports on surgical management for epicardial cysts.

**Case presentation:**

A 73-year-old woman with dyspnea on exertion had a giant cyst (12 × 10 cm in diameter) on preoperative computed tomography. Compression of the left atrium and ventricle by the cyst was considered to be the cause of her symptoms. The cyst was diagnosed with an epicardial cyst intraoperatively. Although the cyst adhered to surrounding tissues, it was successfully resected with off-pump surgery by using a heart positioner and an ultrasonic scalpel.

**Conclusions:**

Surgeons should consider off-pump surgery as an alternative to video-assisted thoracoscopic surgery and on-pump surgery for complicated epicardial cysts.

## Background

Pericardial cysts are uncommon benign tumors, which account for 7% of all mediastinal tumors [1, 2], and they have a reported incidence of 1:100,000 [3–5]. Epicardial cysts are much rarer than pericardial cysts, and surgical management for an epicardial cyst has seldom been reported in the literature. Herein, we report our experience with a giant inflamed epicardial cyst attached to the pulmonary artery and left atrium, which was successfully resected with off-pump surgery.

## Case presentation

**Fig. 1:**
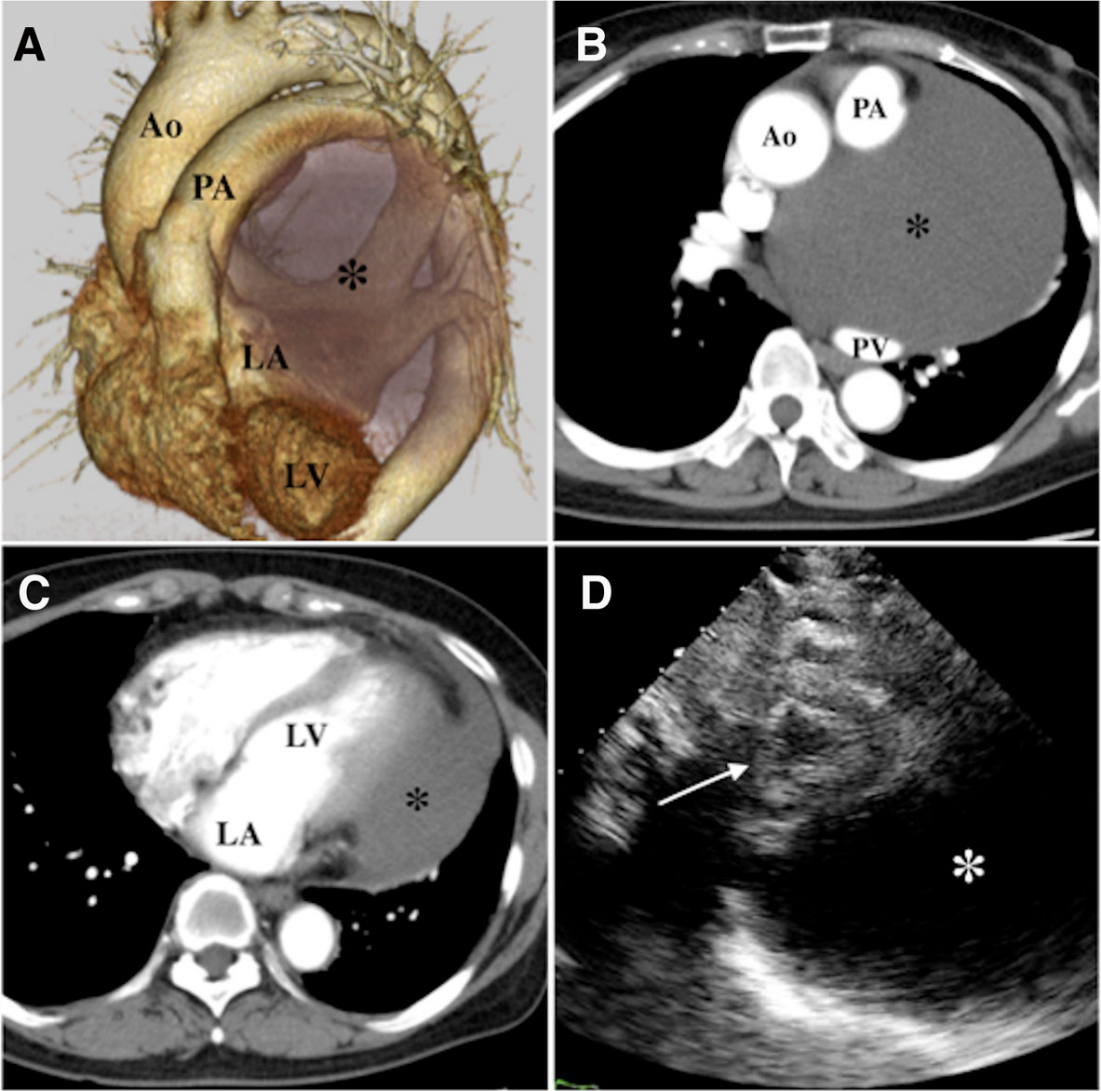
**a** Volume-rendered computed tomography showing a giant cyst (12 × 10 cm in diameter; asterisk) compressing the left atrium and ventricle. Ao aorta, PA pulmonary artery, PV pulmonary vein, LA left atrium, LV left ventricle. **b** Preoperative enhanced computed tomography in the horizontal view showing adhesion of the cyst to the pulmonary artery and pulmonary vein. **c** Computed tomography in the horizontal view showing the cyst compressing the left ventricle. **d** Echocardiography showing the cyst (asterisk) compressing the left ventricle (white arrow)

A 73-year-old woman presented with dyspnea on exertion. Chest radiography showed an enlarged mediastinal silhouette. Preoperative computed tomography (CT) and echocardiography revealed a giant cyst (12 × 10 cm in diameter) occupying a large area around the left atrium and ventricle, and it was present behind the pulmonary artery. Based on the preoperative CT findings, a pericardial or an epicardial cyst was suspected. Additionally, attachment of the cyst to the left atrium, left ventricle, pulmonary artery, and pulmonary vein was suspected (Fig. 1). Compression of the left atrium and ventricle was considered to be the cause of her symptoms. Her hemodynamic condition was stable. Percutaneous cystocentesis was performed, and 800 ml of serous liquid was aspirated. However, 1 week after cystocentesis, the cyst recurred, and its size was the same as that before the procedure. Therefore, resection was planned.

**Fig. 2:**
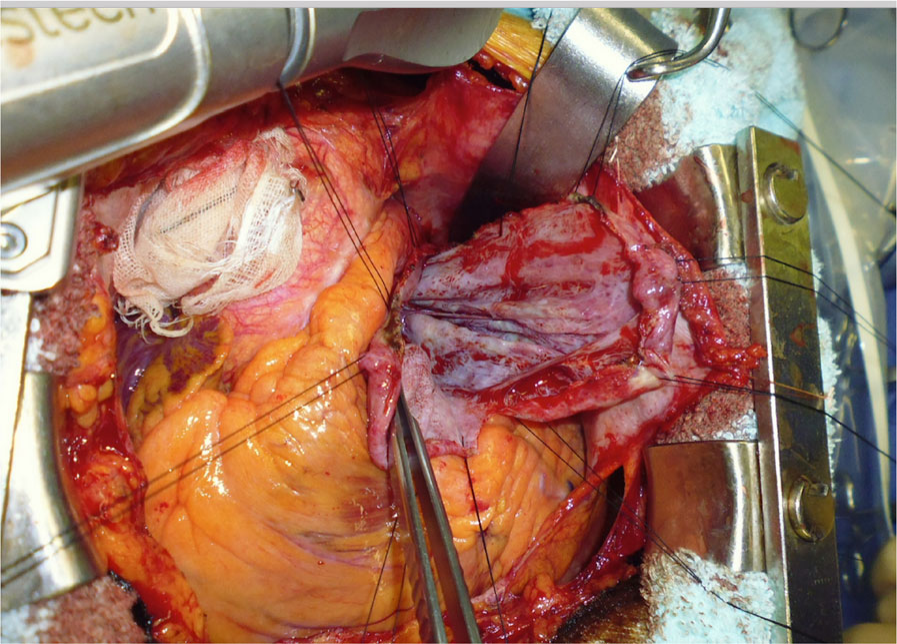
Intraoperative photograph showing the cyst with an unusually thick wall attached to the heart

If the cyst was an epicardial cyst, tight adhesion of the cyst to the left atrium, left ventricle, pulmonary artery, and pulmonary vein was considered possible. However, we believed that the cyst was more likely a pericardial cyst because a pericardial cyst is more common than an epicardial cyst. Therefore, resection involving video-assisted thoracoscopic surgery (VATS) was planned initially. However, the pericardium was intact and an epicardial cyst was diagnosed intraoperatively. As preoperative CT showed compression of the left atrium, ventricle, pulmonary artery, and pulmonary vein, adhesion was suspected. Additionally, the possibility of cardiopulmonary bypass (CPB) was considered. Therefore, open surgery through median sternotomy was performed. The cyst was found to be attached to the visceral pericardium involving the left atrium and pulmonary artery. Contrary to our expectation, the left ventricle was not involved. Additionally, coronary vessels were not involved. The cyst was most tightly attached to the main pulmonary artery. Thus, it was thought to have originated from the pulmonary artery (Fig. 2). The content fluid was aspirated via direct puncture, and a heart positioner was used to provide sufficient traction to the right side, with several sutures placed on the cyst wall, so that the back of the heart and the pulmonary artery could be observed. The cyst wall was thick and hypervascular, and it was completely dissected with an ultrasonic scalpel. The surgery was successfully completed without CPB.

The postoperative course was uneventful. CT confirmed absence of cyst recurrence or pericardial effusion. There has been no recurrence of the cyst since discharge (2 years).

**Fig. 3:**
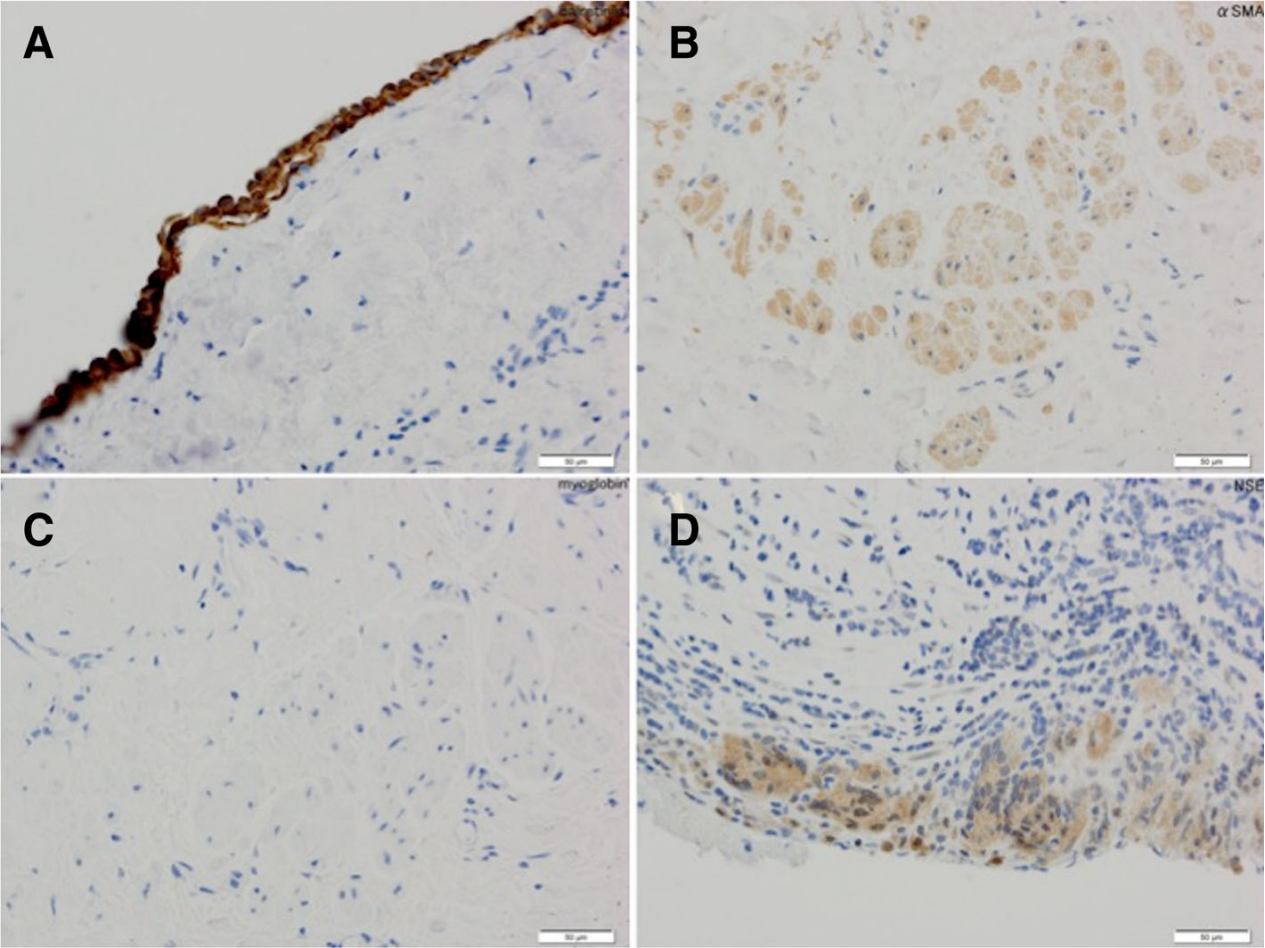
**a** Immunohistochemistry (IHC) for calretinin (bar = 50 μm) showing the presence of mesothelial cells. **b** IHC for alpha-smooth muscle actin (bar = 50 μm) showing the presence of smooth muscles. **c** Negative reactivity of IHC for myoglobin (bar = 50 μm) showing that the smooth muscles did not originate from the myocardium. **d** IHC for a neuron-specific enolase pathologic specimen (bar = 50 μm) showing nerve presence

Histopathological examination revealed a single layer of mesothelial cells. In addition to blood and lymphatic vessels, calretinin-positive cells lining the cystic wall, alpha-smooth muscle actin-positive cells, smooth muscle negative for myoglobin, neuron-specific enolase positive cells, lymphocyte infiltration, and fibrosis were observed (Fig. 3).

## Discussion

This report presented a rare case of an epicardial cyst attached to the left atrium and pulmonary artery. Preoperative differential diagnosis between a pericardial cyst and an epicardial cyst is challenging because they cannot be differentiated on CT [6]. Although MRI is useful to differentiate cysts from other cardiac lesions, it also cannot differentiate between a pericardial cyst and an epicardial cyst. The final diagnosis of a cyst as an epicardial cyst can be made only at surgery [7].

Coronary CT or angiography might be considered to obtain more information, especially with regard to the degree of adhesion and location of the coronary vessels. When coronary CT shows the involvement of coronary vessels, the need for coronary artery bypass grafting or CPB to resect the cyst should be anticipated; however, coronary CT and angiography may not always exclude the possibility of the involvement of coronary vessels preoperatively because some literature reported epicardial cysts with involvement of the coronary vessels and CPB requirement to resect those cysts despite negative findings on coronary CT or angiography [2–4]. Considering the incidence of the disease, we suspected that the cyst was more likely a pericardial cyst than an epicardial cyst. Therefore, involvement of the coronary vessels was not suspected. However, as mentioned above, CT cannot differentiate between a pericardial cyst and an epicardial cyst preoperatively. Moreover, in the present case, CT revealed that the cyst compressed the left ventricle. According to these facts, we should have predicted higher possibility of involvement of the coronary vessels and should have performed coronary CT and angiography for evaluation.

Resection of an epicardial or a pericardial cyst should be considered when the cyst is symptomatic or infected or when it compresses other structures [3, 4]. To prevent these events, some authors suggest that these cysts should be resected even if they are asymptomatic [2–5]. However, surgical management for an epicardial cyst has seldom been discussed. In previous literature, almost all epicardial cysts were resected via median sternotomy. A previous report mentioned that CPB was required when invasion to the ventricular wall or the coronary artery was suspected [4]. VATS has been recently reported as an alternative to median sternotomy for epicardial cysts [8]. If coronary CT or angiography does not show abnormal findings, VATS might be the first option. When invasion to important structures, including the left ventricle and coronary artery, is suspected on preoperative CT, median sternotomy with CPB should be considered. Off-pump resection might be indicated for an epicardial cyst when the left ventricle and coronary artery are not involved.

In our case, on diagnosis of an epicardial cyst during the VATS procedure, we were concerned about adhesion of the cyst to surrounding structures, including the left ventricle, according to preoperative CT findings. In fact, adhesion to the pulmonary artery and left atrium was noted. In addition to median sternotomy, the heart was lifted aside using a heart positioner because the left ventricle was not involved and the cyst could be dissected from the heart using an ultrasonic scalpel without CPB. In hindsight, the cyst could have been resected with VATS; however, the less invasiveness of VATS was not considered beneficial enough to outweigh the risks associated with increased surgical complexity.

## Conclusions

Our case report highlights the feasibility of off-pump surgical resection for a complicated epicardial cyst. Adhesion of a cyst to surrounding tissues may make resection with VATS difficult. Although this condition is rare, surgeons should keep in mind that off-pump surgery is an alternative to VATS and on-pump surgery for an epicardial cyst attached to surrounding tissues.

## Abbreviations

CPB: Cardiopulmonary bypass
CT: Computed tomography
MRI: Magnetic resonance imaging
VATS: Video-assisted thoracoscopic surgery
